# Nrf2 Regulates the Risk of a Diesel Exhaust Inhalation-Induced Immune Response during Bleomycin Lung Injury and Fibrosis in Mice

**DOI:** 10.3390/ijms18030649

**Published:** 2017-03-17

**Authors:** Ying-Ji Li, Takako Shimizu, Yusuke Shinkai, Yukiyo Hirata, Hirofumi Inagaki, Ken Takeda, Arata Azuma, Masayuki Yamamoto, Tomoyuki Kawada

**Affiliations:** 1 Department of Hygiene and Public Health, Nippon Medical School, Tokyo 113-0031, Japan; takako-s@nms.ac.jp (T.S.); yuki-hir@nms.ac.jp (Y.H.); hrfmi@nms.ac.jp (H.I.); kawada@nms.ac.jp (T.K.); 2 The Center for Environmental Health Science for the Next Generation, Research Institute for Science and Technology, Tokyo University of Science, Noda 278-8510, Japan; shinkai49@gmail.com (Y.S.); takedak@rs.noda.tus.ac.jp (K.T.); 3 Department of Pulmonary Medicine/Infection and Oncology, Nippon Medical School, Tokyo 113-8602, Japan; azuma_arata@yahoo.co.jp; 4 Department of Biochemistry, Tohoku University Graduate School of Medicine, Sendai 980-8575, Japan; masiyamamoto@med.tohoku.ac.jp

**Keywords:** diesel exhaust, bleomycin, lung injury and fibrosis, Nrf2, oxidative stress/antioxidative stress

## Abstract

The present study investigated the effects of diesel exhaust (DE) on an experimental model of bleomycin (BLM)-induced lung injury and fibrosis in mice. BLM was intravenously administered to both *Nrf2*^+/+^ and *Nrf2*^−/−^ C57BL/6J mice on day 0. The mice were exposed to DE for 56 days from 28 days before the BLM injection to 28 days after the BLM injection. Inhalation of DE induced significant inhibition of airway clearance function and the proinflammatory cytokine secretion in macrophages, an increase in neutrophils, and severe lung inflammatory injury, which were greater in *Nrf2*^−/−^ mice than in *Nrf2*^+/+^ mice. In contrast, inhalation of DE was observed to induce a greater increase of hydroxyproline content in the lung tissues and significantly higher pulmonary antioxidant enzyme mRNA expression in the *Nrf2*^+/+^ mice than in *Nrf2*^−/−^ mice. DE is an important risk factor, and Nrf2 regulates the risk of a DE inhalation induced immune response during BLM lung injury and fibrosis in mice.

## 1. Introduction

Epidemiological studies suggest that air pollutants, including diesel exhaust (DE), may have contributed to the recent rises in the morbidity and mortality rates of respiratory conditions [1,2]. The reactive oxygen species (ROS) generated in response to DE exposure and the subsequent initiation of the oxidative stress response have been suggested to play important roles in the adverse effects of DE particles (DEP) in in vitro experimental studies [3,4,5,6,7,8]. Furthermore, our previous in vivo studies indicated that antioxidant response elements may determine the host’s susceptibility to the adverse effects of DE [9,10,11].

Human idiopathic pulmonary fibrosis (IPF) is a progressive and fatal disorder [12,13]. Although the precise mechanisms of IPF are not fully understood, the oxidant/antioxidant balance may play an important role in many of the associated inflammatory and fibrotic processes [14]. Recent studies have also indicated that the antioxidant *N*-acetylcysteine improves IPF [15]. Bleomycin (BLM) causes oxidative lung damage accompanied by excess ROS generation, resulting in the oxidation of cellular macromolecules (e.g., lipid peroxidation), DNA breakage, and pulmonary fibrosis [16]. 

The nuclear factor erythroid-derived 2-like 2 (Nfe2l2), which is also known as NF-E2-related factor 2 (Nrf2), is a transcription factor that is essential for the induction and/or constitutive expression of phase II and antioxidant enzymes [17]. Nrf2 is particularly important for protecting cells and tissues in highly oxidative microenvironments, including the airway, which interfaces with the external environment and is exposed to pollutants and other oxidant stressors [18]. Our previous study suggested that oxidative stress is involved in DE-induced airway inflammation [11] and allergic asthma [19], as evidenced by experiments involving *Nrf2* knockout mice. Nrf2 regulates antioxidant defense, which is the main defensive process against the proinflammatory and oxidizing effects of DEP [8]. 

Air pollution, such as by DE, has recently attracted considerable attention [20]. It has been reported that the maximum hourly concentration of atmospheric particles with a diameter of ≤2.5 μm can reach 701 μg/m^3^ in some urban environments [21]. Many reports have suggested that DE exacerbates the onset and progression of allergic asthma at both high (DEP: 1–3 mg/m^3^) [22,23] and low concentrations (DEP: 100 μg/m^3^) [19]. However, the effects of DE on the pathogenesis of pulmonary fibrosis are unclear. We hypothesized that DE is a risk factor for pulmonary fibrosis caused by oxidative stress, particularly in the early phase of lung inflammation, which may cause acute exacerbations. This study was designed to (1) confirm the effects of DE (DEP: 1 mg/m^3^, which is similar to the concentrations that are inhaled in urban environments with serious air pollution) in an experimental model of BLM-induced lung injury and fibrosis; and (2) to identify the molecular mechanisms involved in these effects using wild-type mice (*Nrf2*^+/+^) and *Nrf2*-knockout mice (*Nrf2*^−/−^). 

## 2. Results

### 2.1. Differential Cell Counts in Bronchoalveolar Lavage (BAL) Fluid (BALF)

**Figure 1:**
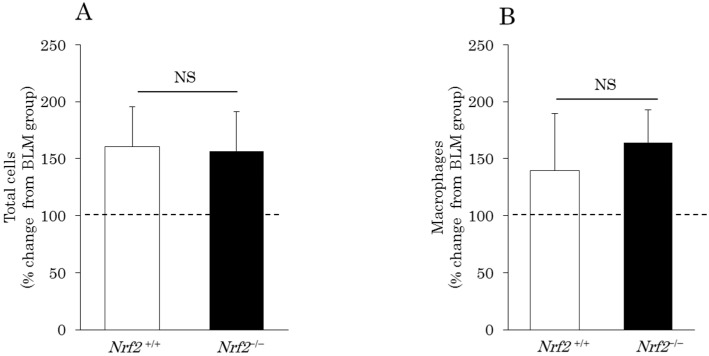

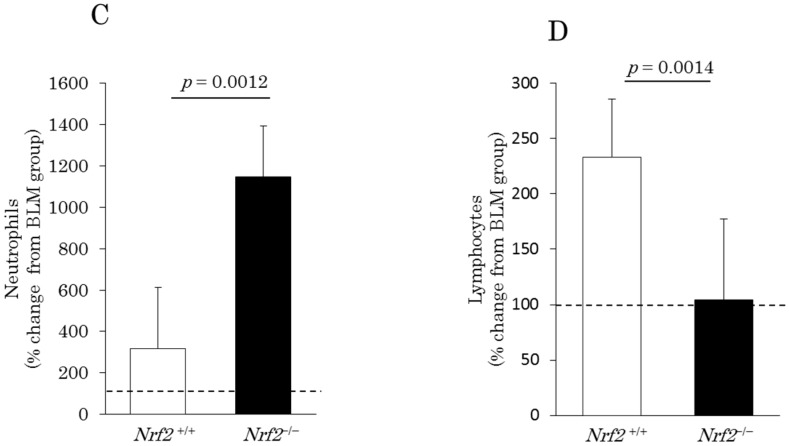
Differential cell counts in the bronchoalveolar lavage fluid (BALF) on day 10 after bleomycin (BLM) injection and after exposure to diesel exhaust (DE) for 38 days. The vertical axis shows percentage changes in the cell numbers of the DE plus BLM group relative to the BLM group. (**A**) total cells; (**B**) macrophages; (**C**) neutrophils; (**D**) lymphocytes. Data are shown as mean ± standard deviation (SD) values in each group (*n* = 5). NS: no significant difference.

In the BLM-induced lung injury model, the percentage changes in the BALF cells, total cells (Figure 1A), macrophages (Figure 1B), and neutrophils (Figure 1C) markedly increased after DE exposure in both the *Nrf2*^+/+^ mice (total cells: 160.2% ± 35.6%; macrophages: 139.2% ± 50.5%; neutrophils: 316% ± 298%) and *Nrf2*^−/−^ mice (total cells: 156.6% ± 34.4%; macrophages: 164% ± 28.7%; neutrophils: 1147% ± 245.7%) relative to the BLM group. The increase in the macrophage count induced by DE was slightly higher in the *Nrf2*^−/−^ mice than in the *Nrf2*^+/+^ mice, however, the difference is not significant (Figure 1B). The increase in the neutrophil count induced by DE was significantly greater in the *Nrf2*^−/−^ mice than in the *Nrf2*^+/+^ mice (Figure 1C). In contrast, the percentage changes in the lymphocytes (Figure 1D), relative to the BLM group, markedly increased after DE exposure only in the *Nrf2*^+/+^ mice (233.4% ± 52.1%); no marked change was found after DE exposure in the *Nrf2*^−/−^ mice (104% ± 73.6%). 

### 2.2. DEP-Laden Alveolar Macrophages 

**Figure 2:**
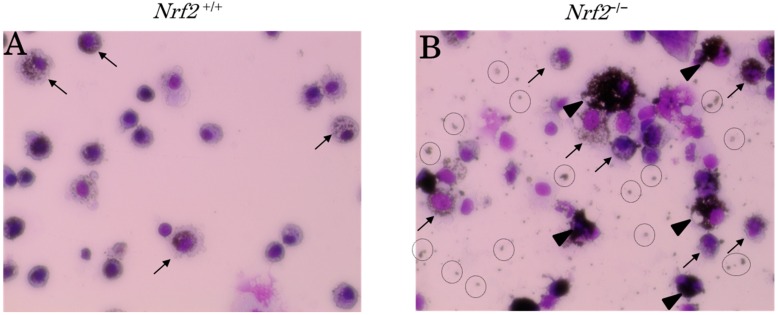
Representave optical micrographs of May-Gimza (Kobe, Japan) stained cell preparation (Original × 400). The pathologic features of diesel exhaust (DE) particles (DEP)-laden alveolar macrophages in the BALF on day 10 after BLM injection and after exposure to DE for 38 days. (**A**) DE plus BLM-treated group in *Nrf2*^+/+^ mice; (**B**) DE plus BLM-treated group in *Nrf2*^−/−^ mice. Arrows indicate DEP-laden alveolar macrophages. Arrowhead indicates coal-black alveolar macrophages. Dotted ring indicates DEP in the extracellular background of the cytopreparation.

**Figure 3:**
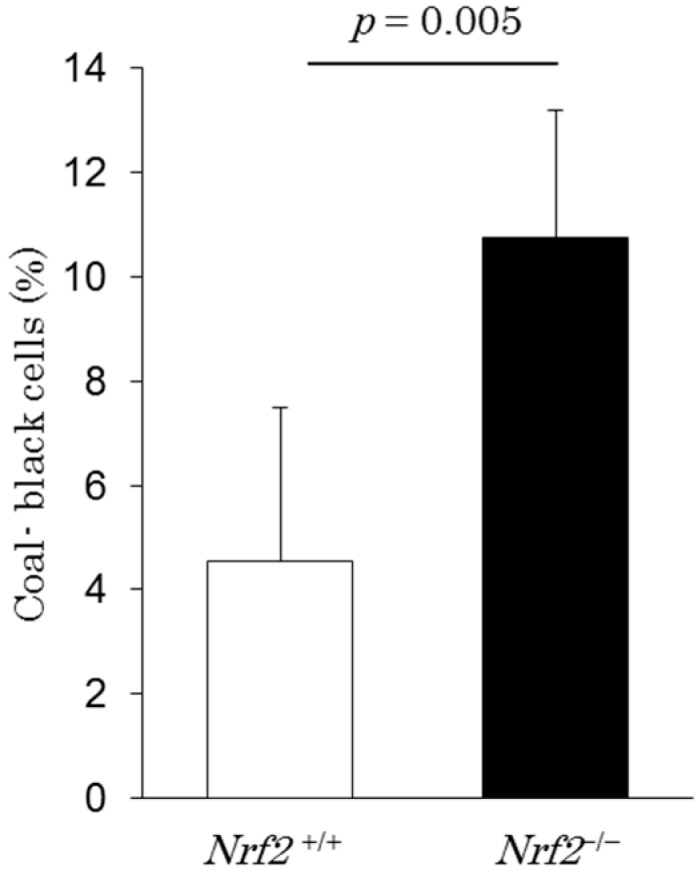
The coal-black alveolar macrophages in the BALF on day 10 after BLM injection and after exposure to DE for 38 days. The vertical axis shows percentage of coal-black alveolar macrophages, in which DEP accounted for more than half of the cytoplasm, such as those indicated in Figure 2B (arrowhead), in the total DEP-laden alveolar macrophages. Data are shown as mean ± SD values in each group (*n* = 5).

We assessed the DEP-laden alveolar macrophages in the BALF of the DE exposure groups. Typical pictures of DEP-laden alveolar macrophages (arrows) are shown in Figure 2. It was evident that most alveolar macrophages engulfed DEP in both the *Nrf2*^+/+^ and *Nrf2*^−/−^ mice in the DE exposure groups. However, the DEP content of each macrophage was markedly lower in *Nrf2*^+/+^ mice (Figure 2A) than in *Nrf2*^−/−^ mice (Figure 2B), and the backgrounds of the pictures were clearer in *Nrf2*^+/+^ mice (Figure 2A) than in *Nrf2*^−/−^ mice (Figure 2B). The DEP-laden alveolar macrophage counts were performed in ten separate fields per smear. The percentage of coal-black alveolar macrophages, in which DEP accounted for more than half of the cytoplasm, such as those indicated in Figure 2B (arrowhead), in the total DEP-laden alveolar macrophages was significantly higher in *Nrf2*^−/−^ mice than in *Nrf2*^+/+^ mice (Figure 3). 

### 2.3. MIP-2, TNF-α, and TGF-β1 Concentrations in BALF

**Figure 4:**
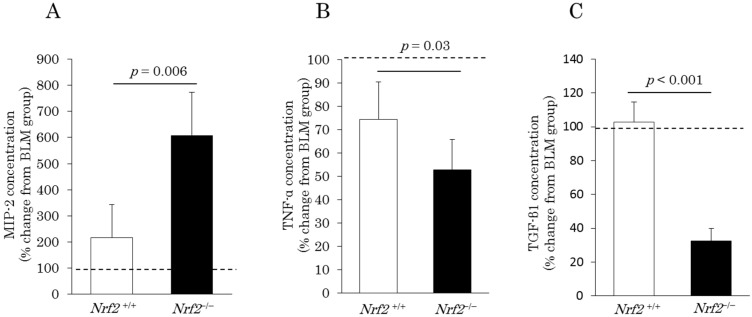
Macrophage inflammatory protein (MIP)-2 (**A**); tumor necrosis factor (TNF)-α (**B**); and transforming growth factor (TGF)-β1 (**C**) concentrations in the BALF on day 10 after BLM injection and after exposure to DE for 38 days. The vertical axis shows percentage changes in the cytokines of the DE plus BLM group relative to the BLM group. Data are shown as mean ± SD values in each group (*n* = 5).

The percentage changes in the concentration of C-X-C motif chemokine ligand 2 (CXCL2) or also known as macrophage inflammatory protein (MIP)-2, relative to the BLM group, markedly increased after DE exposure in both the *Nrf2*^+/+^ mice (216.2% ± 128.1%) and *Nrf2*^−/−^ mice (608.7% ± 165.3%). The increase in the concentration of MIP-2 induced by DE was significantly higher in the *Nrf2*^−/−^ mice than in the *Nrf2*^+/+^ mice (Figure 4A). In contrast, the percentage changes in the concentration of tumor necrosis factor (TNF)-α, relative to the BLM group, decreased markedly after DE exposure in both the *Nrf2*^+/+^ mice (74.4% ± 16.1%) and *Nrf2*^−/−^ mice (52.7% ± 13%); the decrease in the concentration of TNF-α induced by DE was significantly greater in the *Nrf2*^−/−^ mice than in the *Nrf2*^+/+^ mice (Figure 4B). The percentage changes in the transforming growth factor (TGF)-β1 concentration (Figure 4C), relative to the BLM group, decreased significantly after DE exposure only in the *Nrf2*^−/−^ mice (32.6% ± 7.2%); no marked change was found after DE exposure in the *Nrf2*^+/+^ mice (102.7% ± 11.9%). 

### 2.4. Induction of Pulmonary Antioxidant Enzyme mRNA Expression 

**Figure 5:**
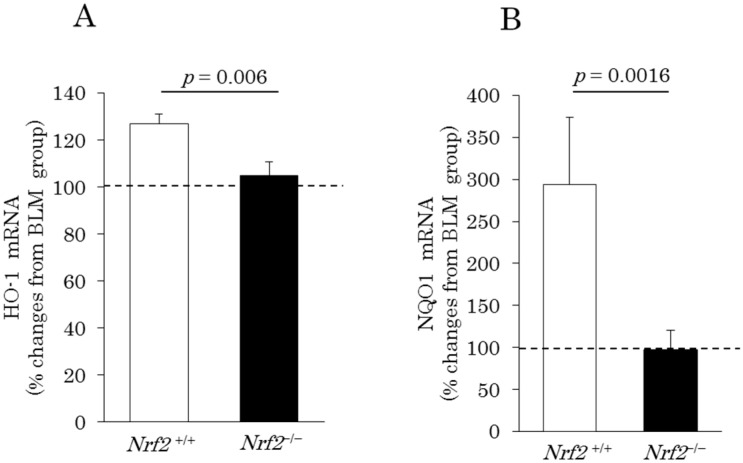
Heme oxygenase (HO)-1 (**A**); and NAD(P)H quinone dehydrogenase (NQO)1 (**B**) mRNA expression levels in the lung tissues on day 10 after BLM injection and after exposure to DE for 38 days. The vertical axis shows percentage changes in the target gene mRNA expression levels of the DE plus BLM group relative to the BLM group. β-actin was used as an internal control. Data are shown as mean ± SD values in each group (*n* = 3–5).

The changes in the pulmonary mRNA expression levels of heme oxygenase (HO)-1 and NAD(P)H quinone dehydrogenase (NQO)1 observed after DE exposure were determined by quantitative real-time reverse transcription-polymerase chain reaction. After DE exposure, percentage changes in pulmonary heme oxygenase (HO)-1 mRNA expression of 126.7% ± 4.2% and 104.7% ± 5.9%, compared with the values seen in the BLM group, were observed in the *Nrf2*^+/+^ and *Nrf2*^−/−^ mice, respectively (Figure 5A). In addition, percentage changes in pulmonary NQO1 mRNA expression of 294.1% ± 79.6% and 96.9% ± 23.3%, compared with the values recorded in the BLM group, were found in the *Nrf2*^+/+^ and *Nrf2*^−/−^ mice, respectively (Figure 5B). Thus, the pulmonary mRNA expression levels of antioxidant enzymes HO-1 and NQO1 were significantly higher in the *Nrf2*^+/+^ mice than in the *Nrf2*^−/−^ mice.

### 2.5. Histopathologic Assessment

**Figure 6:**
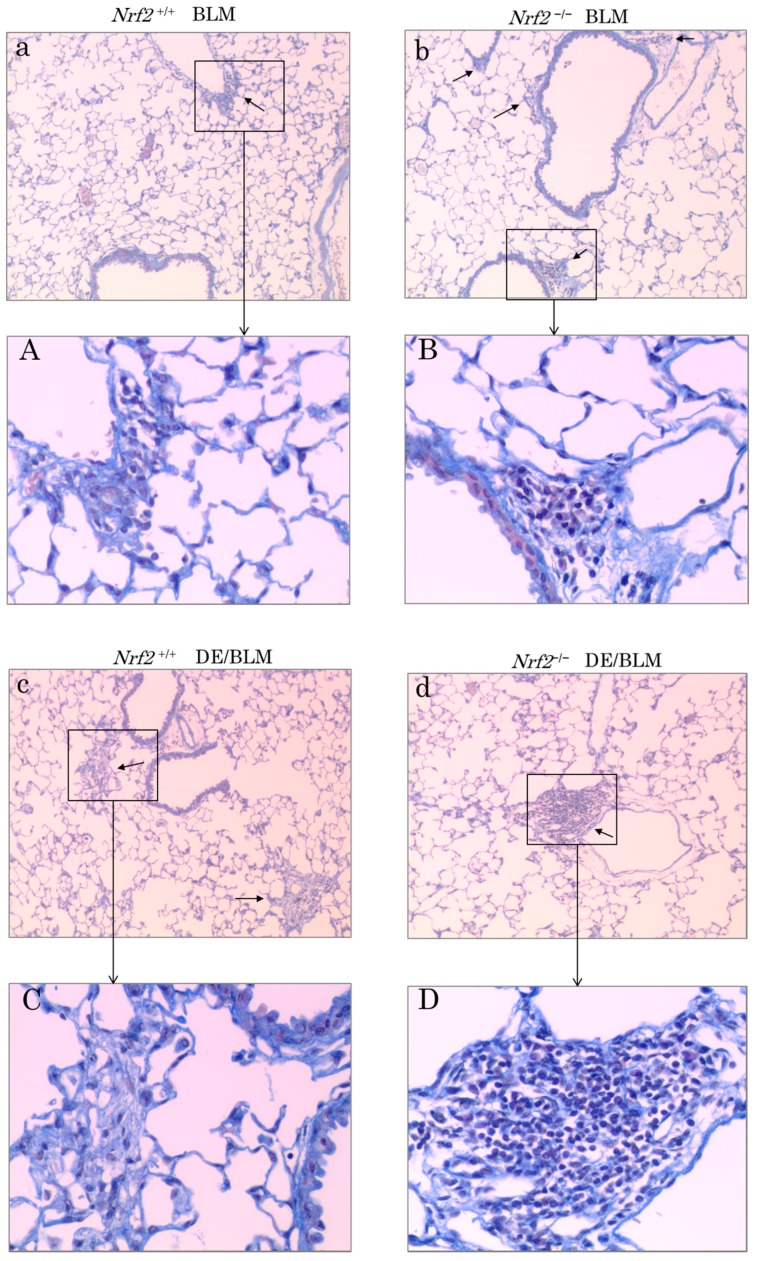

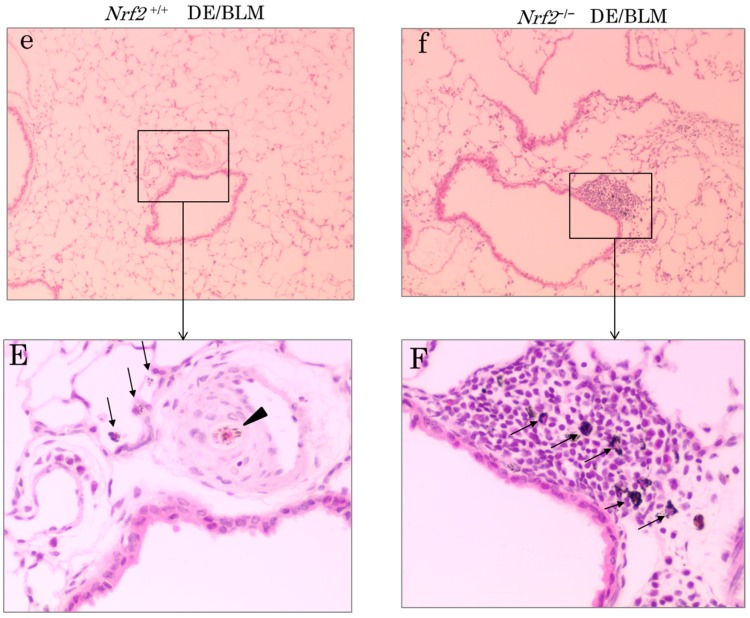
Histopathologic assessment on day 28 after BLM injection and after exposure to DE for a maximum of 56 days. These photographs show typical results. Clean air plus BLM-treated group in *Nrf2*^+/+^ mice (**A**,**a**) and *Nrf2*^−/−^ mice (**B**,**b**); DE plus BLM-treated group in *Nrf2*^+/+^ mice (**C**,**c**) and *Nrf2*^−/−^mice (**D**,**d**). (**A**–**D**) original ×400; (**a**–**d**) original ×100, Masson Trichrome stain (MT) stain. The collagens fibers are blue colored in MT stain and triangle-headed arrows indicate fibroblastic foci. DE plus BLM-treated group in *Nrf2*^+/+^ mice (**E**,**e**) and *Nrf2*^−/−^ mice (**F**,**f**). (**E**,**F**) original ×400; (**e**,**f**) original ×100, Hematoxylin Eosin (HE) stain. Arrows indicate DEP-laden alveolar macrophages surrounding the lesion. Arrowhead indicates the accumulated DEP-laden alveolar macrophages, which was considered a foreign-body giant cell (**E**).

Tissue sections were prepared from mouse left lung tissues and observed for histological changes by light microscopy at 28 days after BLM administration and after exposure to DE for a maximum of 56 days. Typical pictures of the lung histopathologic assessment are shown in Figure 6. Pulmonary fibroblastic foci induced by BLM injection were evident in all groups. In the BLM lung injury model without DE exposure, pulmonary fibroblastic foci increased more in *Nrf2*^−/−^ mice (Figure 6b,B) than in *Nrf2*^+/+^ mice (Figure 6a,A). In *Nrf2*^+/+^ mice, pulmonary fibroblastic foci increased more in the BLM plus DE exposure group (Figure 6c,C) than in the group without DE exposure (Figure 6a,A); in *Nrf2*^−/−^ mice, the peribronchial and perivascular infiltration of cells increased more in the BLM plus DE exposure group (Figure 6d,D) than in the group without DE exposure (Figure 6b,B). The peribronchial and perivascular infiltration of cells in the BLM plus DE exposure group increased more in *Nrf2*^−/−^ mice (Figure 6d,D) than in *Nrf2*^+/+^ mice (Figure 6c,C). The peribronchial infiltration of cells and of DEP-laden alveolar macrophages (arrows) in *Nrf2*^−/−^ mice (Figure 6f,F) was higher than in *Nrf2*^+/+^ mice (Figure 6e,E). In the *Nrf2*^+/+^ mice, epithelioid-like cell hyperplasia was present around the accumulated DEP-laden alveolar macrophages, which was considered a foreign-body giant cell (Figure 6E, arrowhead). However, the same observation was not found in the *Nrf2*^−/−^ mice.

### 2.6. Hydroxyproline Content in Lung Tissues

**Figure 7:**
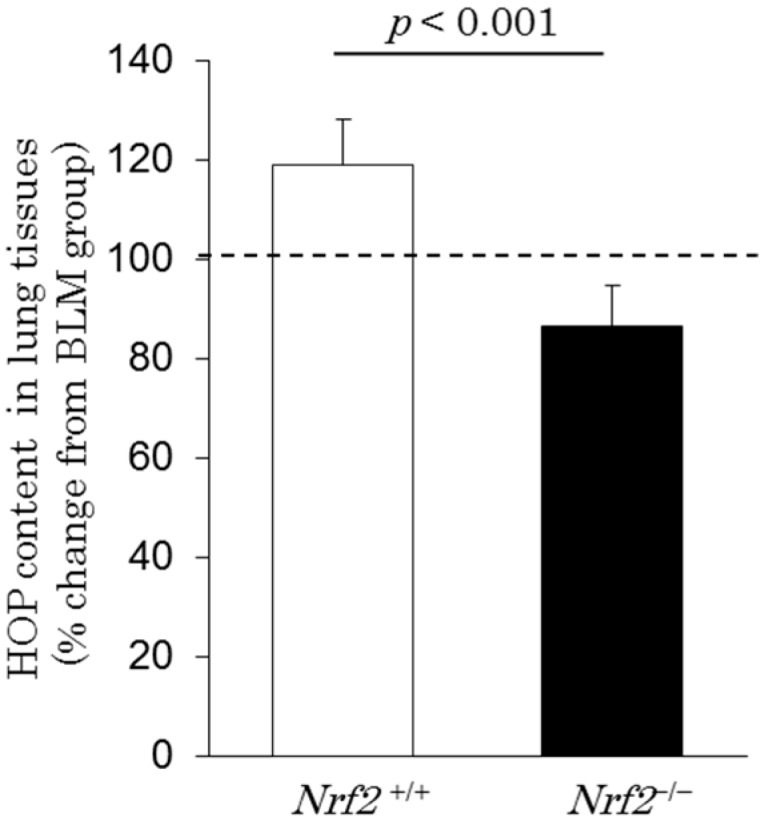
Hydroxyproline (HOP) content in the lung tissues on day 28 after BLM injection and after exposure to DE for a maximum of 56 days. The vertical axis shows percentage changes in the HOP content of the DE plus BLM group relative to the BLM group. Data are shown as mean ± SD values in the *Nrf2*^+/+^ group (*n* = 5) and *Nrf2*^−/−^ group (*n* = 6).

Pulmonary fibroblastic foci induced by bleomycin injection were evident in all groups. To evaluate the severity of interstitial fibrosis among the groups, the hydroxyproline (HOP) content in the right lung tissues was analyzed on day 28 after BLM administration and after exposure to DE for a maximum of 56 days in all groups. The percentage changes in the HOP content, relative to the BLM group, were increased in *Nrf2*^+/+^ mice (119% ± 9%), but decreased in *Nrf2*^−/−^ mice (86% ± 8%) after DE exposure (Figure 7). 

## 3. Discussion 

This study evaluated the mechanisms involved in the development of DE induced pulmonary damage in a BLM-induced lung injury and fibrosis model using both *Nrf2*^+/+^ and *Nrf2*^−/−^ mice. The present study first demonstrated that in the BLM-induced lung injury and fibrosis model inhalation of DE increased the HOP content in the lung tissues in *Nrf2*^+/+^ mice to a greater extent than in *Nrf2*^−/−^ mice; inhalation of DE also induced granulomas in the lung tissues to eliminate the intracellular DEP observed only in *Nrf*2^+/+^ mice. In contrast, significantly greater inhibition of alveolar macrophage function and severe lung inflammatory injury were induced by inhalation of DE in *Nrf2*^−/−^ mice than in *Nrf2*^+/+^ mice. Nrf2 is an important factor for regulating the risk of DE inhalation induced immune response during BLM-induced lung injury and fibrosis in mice.

One advantage of the intravenous administration of BLM is that it closely mimics the way in which humans are exposed to the drug [24]. Therefore, in our study, BLM was administered intravenously to induce pulmonary inflammation followed by fibrosis in mice, as reported previously [25]. It is known that Nrf2 has a critical role in protection against pulmonary fibrosis, presumably through enhancement of cellular antioxidant capacity [26,27]. In the presented study, to evaluate the effects of DE exposure in BLM-induced lung injury and fibrosis in both *Nrf2*^+/+^ and *Nrf2*^−/−^ mice, respectively, the percentage changes relative to the BLM group (clean air) in *Nrf2*^+/+^ and *Nrf2*^−/−^ mice, respectively, were used for the data analysis.

Although the pathogenesis of pulmonary fibrosis remains unclear, many investigators have found that innate immune cells, particularly neutrophils and macrophages, may play a role in lung fibrosis. The activation of macrophages occupies a pivotal role in the translation of injury to aberrant repair in IPF, but the reasons for macrophage accumulation in lung fibrosis are not always clear [28,29]. Migration of neutrophils to the site of injury allows them to exhibit a range of functions, including the release of neutrophil elastase (NE), where they may then impact the fibrotic process. NE promotes fibroblast proliferation and myofibroblast differentiation in vitro, and mice deficient in NE do not develop asbestos-induced pulmonary fibrosis [30]. Our previous study showed that the number of macrophages, neutrophils, and lymphocytes in the BALF significantly increased during the early period after the intravenous administration of BLM [25].

DEP are carbon-based particles that adsorb various organic compounds, including polycyclic aromatic hydrocarbons (PAHs), quinones, and nitro-PAHs. Both the organic and particulate components play a role in DE-induced pulmonary toxicity [31]. It is well known that alveolar macrophages play an important role in the first defense against various environmental particles and microorganisms. Inhalation exposure of rats to DE suppresses alveolar macrophage phagocytic function and their secretion of proinflammatory cytokines [32,33]. Human challenge studies with DE have detected increased neutrophilic inflammation in the airway [34,35].

In the present study, the DEP content of the macrophages and the extracellular DEP in the BALF were remarkably higher in *Nrf2*^−/−^ mice than in *Nrf2*^+/+^ mice, suggesting that Nrf2 is involved with the attenuation of the airway clearance function by DE exposure in the BLM lung injury model. During the early phase in the BLM lung injury model of *Nrf2*^−/−^ mice, DE exposure suppressed the content of TNF-α and TGF**-**β in the BALF, suggesting that the proinflammatory cytokine secretion function in macrophages is also attenuated by the DE exposure-mediated Nrf2 pathway in the BLM lung injury model. This would be consistent with the observation that the activation of alveolar macrophage antioxidant defenses is mediated through Nrf2 and its downstream effectors [36]. Suppressed macrophage function may affect antigen presentation to lymphocytes; therefore, lymphocytes did not respond to DE exposure in *Nrf2*^−/−^ mice, and the promoted neutrophil recruitment in *Nrf*2^−/−^ mice mediated increased MIP-2 in the present study. MIP2 is known as C-X-C motif chemokine 2 (CXCL2) and has been shown to stimulate the migration and activation of neutrophils [37]; neutralization of MIP2 attenuates bleomycin-induced pulmonary fibrosis [38]. In the *Nrf2*^−/−^ mice, the neutrophils in the BALF increased about 10-fold over the BLM group after DE exposure. Neutrophil extracellular traps are released by neutrophils and cause local tissue damage and inflammation [39,40]. In human IPF patients, the presence of neutrophils is significant and a doubling of neutrophils, specifically in lavage fluid at baseline, is a predictor of early death [41]. In the BLM lung injury model, the histological finding showed that the peribronchial and perivascular infiltration of cells was clearly greater in the *Nrf2*^−/−^ mice than in *Nrf2*^+/+^ mice after DE exposure. The severe lung inflammatory injury in the lung tissues after DE exposure in the *Nrf2*^−/−^ mice is considered to be caused by neutrophilic lung inflammation. 

In contrast, the BLM lung injury model in *Nrf2*^+/+^ mice exhibited significantly higher pulmonary mRNA expression levels of the antioxidant enzymes HO-1 and NQO-1 than in the *Nrf2*^−/−^ mice after DE exposure. Cytoprotective pathways involving the production of antioxidant enzymes are induced by the Nrf2 signaling pathway at minimal levels of oxidative stress, and this may constitute the first tier of the hierarchical oxidative stress response. If these enzymes fail to neutralize the effects of ROS, proinflammatory effects constitute a second tier of the oxidative stress response; the superimposed level of oxidative stress results in cytotoxicity, including the initiation of programmed cell death [6]. A recent report suggests that human bronchial epithelial cells exposed in vitro to DEP exhibit indicators associated with decreases in antioxidant defenses and imbalances in pro- and anti-apoptotic gene expression [42]. Our results indicate that Nrf2 deficiency results in decreased HOP content in the lung tissues after DE exposure in the BLM lung injury model. This finding suggests that in *Nrf2*^−/−^ mice, severe oxidative damage induces apoptosis of epithelial cells, therefore maybe unable to development and progress of fibrosis.

Interestingly, in the BLM lung injury model in *Nrf2*^+/+^mice, we found a giant cell-like granuloma formation caused by DE exposure; however, a similar phenomenon was not observed in the *Nrf2*^−/−^mice. Granulomas are a collection of immune cells known as macrophages [43], and form as an immune reaction to a foreign substance. Our results suggest that in the BLM lung injury model in *Nrf2*^+/+^mice, their ability to fuse to form giant cells that coalesce into granulomas in response to inflammatory stimuli is important in the elimination of intracellular DEP and may be under the regulation of the host antioxidant defense function mediated by the Nrf2 signal pathway. This mechanism would be consistent with the report of a significant reduction of granulomas in *Nrf2*-deficient mice infected with mycobacterium tuberculosis [44]. 

Furthermore, in *Nrf2*^+/+^ mice, pulmonary fibroblastic foci in histological findings and HOP content in the lung tissues increased more in the BLM plus DE exposure group than in the group without DE exposure, however, no marked change in TGF-β in the BALF was found after DE exposure in the *Nrf2*^+/+^ mice. In the *Nrf2*^+/+^ mice, the neutrophils also increased about 3fold over the BLM group after DE exposure, although the increase was significantly lower than in *Nrf2*^−/−^ mice. Neutrophils release NE, which promotes fibroblast proliferation and myofibroblast differentiation in vitro and in mice. NE may promote TGF-β activation, but can induce myofibroblast differentiation independently of TGF-β [30]. The inhalation of DE increased the HOP content in the lung tissues in *Nrf2*^+/+^ mice independently of TGF-β in the present study; this result may be caused by similar mechanisms, which is a subject for future analysis. Recent research also demonstrates that the progression from granuloma to fibrosis begins with persistent, uncontrolled inflammation and is associated with pro-fibrotic genetic features and immune responses [45].

In the BLM lung injury model without DE exposure, both the HOP content in the lung tissues (Figure S2) and the TGF-β level in the BALF (Figure S1) increased significantly more in *Nrf2*^−/−^ mice than in *Nrf2*^+/+^ mice. Pulmonary fibroblastic foci induced by BLM injection without DE exposure also increased more in *Nrf2*^−/−^ mice than in *Nrf2*^+/+^ mice. These results are consistent with previous reports [26]. 

These findings show that in the BLM lung injury model responses to DE exposure may cause an increase in lung fibrosis in the *Nrf2*^+/+^ mice and cause severe lung inflammatory injury in the *Nrf2*^−/−^ mice. DE is an important risk factor, and Nrf2 regulates the risk of a DE inhalation-induced immune response during BLM lung injury and fibrosis in mice. Our results suggest that living in areas with air pollution, such as high levels of DE exposure, may cause acute exacerbation in individuals with lung fibrosis due to defective Nrf2 genotypes. Targeting Nrf2 signaling may attenuate the risk from DE exposure in populations susceptible to IPF.

## 4 Materials and Methods

### 4.1. Animals

Wild-type *(Nrf2*^+/+^) C57BL/6 mice were purchased from CLEA Japan (Tokyo, Japan). *Nrf2* knockout (*Nrf2*^−/−^) C57BL/6 mice were initially obtained from RIKEN BRC (RBRC No. 01390, Tsukuba, Japan) and backcrossed onto the C57BL/6 background in our laboratory. *Nrf2*^−/−^ C57BL/6 mice were generated as described previously [17]. The mice were genotyped for Nrf2 via the PCR-based amplification of genomic DNA extracted from the tail, as described previously [11]. Briefly, PCR amplification was performed using the following three primers: *Nrf2*-sense for both genotypes: 5′-TGGACGGGACTATTGAAGGCTG-3′; *Nrf2*-antisense for the wild-type mice: 5′-GCCGCCTTTTCAGTAGATGGAGG-3′; *Nrf2*-antisense for LacZ: 5′-GCGGATTGACCGTAATGGGATAGG-3′.

The amplification conditions involved 30 cycles of 96 °C for 20 s, 59 °C for 30 s, and 72 °C for 45 s. The wild-type allele produces a 734-bp band, whereas the knockout allele produces a 449-bp band. The mice used in this study were 6–8 weeks old and were housed under specific pathogen-free conditions. All procedures were approved by the Animal Care and Use Committee and the Genetic Modification Safety Committee of Nippon Medical School (approval code: 21-7). The mice were randomly classified into each experimental group and housed in wire-mesh cages in clean air or a DE exposure chamber (Nanoparticles Health Science Research Center, Tokyo University of Science, Noda-shi, Japan). All procedures were approved by Tokyo University of Science’s Animal Care and Use Committee. 

### 4.2. DE Exposure

The mice were exposed to DE in an inhalation chamber at the Nanoparticles Health Science Research Center, Tokyo University of Science, according to the method described in previous reports [46,47]. Briefly, a 2179 L, 39 Kw/3000 rpm diesel engine (Isuzu Motors Ltd., Tokyo, Japan) was used. The mass and concentrations of DEP were measured using a Piezobalance dust monitor (model 3521; Kanomax Inc., Osaka, Japan) and a condensation particle counter (model 3007; TSI Inc., Shoreview, MN, USA), respectively. The concentrations of gas components (nitric oxide (NO_x_), sulfur dioxide (SO_2_), and carbon monoxide (CO)) in the chambers were measured using an NO-NO_2_-NO_x_ analyzer (model 42i, Thermo Fisher Scientific Inc., Franklin, MA, USA), an enhanced trace level SO_2_ analyzer (model 43i-TLE, Thermo Fisher Scientific Inc.), and a CO analyzer (model 48i, Thermo Fisher Scientific Inc.). The concentration of DEP in the DE gas was adjusted to approximately 1 mg/m^3^. The mean concentration of DE is shown in Table 1. The mice were exposed to DE for 8 h/day and 6 days/week.

### 4.3. Study Design

The *Nrf2*^+/+^ and *Nrf2*^−/−^ mice were divided into the clean air plus BLM (BLM group) and DE exposure plus BLM (DE/BLM group) groups. Mice were exposed to DE or clean air for a maximum of 56 days. BLM (Nippon Kayaku, Tokyo, Japan) was dissolved in normal saline solution (NS; Otsuka Pharmaceutical Co., Ltd., Tokyo, Japan) and intravenously administered to the mice at a dosage of 80 mg/kg body weight, as identified in a preliminary experiment (data not shown), and 0.3 mL per mouse [25]. BLM was intravenously administered to the *Nrf2*^+/+^ and *Nrf2*^−/−^ mice after they had been exposed to clean air or DE for 28 days (day 0). The mice in all groups were sacrificed at 10 days or 28 days after the BLM injection and on the last day of DE exposure (Supplementary Figure S3). Ten days after BLM injection, all groups were examined for BALF and for induction of mRNA of target genes in lung tissues. Twenty-eight days after BLM injection, all groups were examined for histopathological features and HOP measurement of the lung tissues. The DEP exposure concentration and time, BLM concentration per mouse, and the timing of performing BAL were based on preliminary experiment data (data not shown).

### 4.4. Histopathological Assay

Mice were sacrificed by an intraperitoneal injection with an overdose of pentobarbital (Somnopentyl, Kyoritsu Seiyaku Corporation, Tokyo, Japan). For histological examination, 10% formalin (Wako, Osaka, Japan) fixed left lung tissues in all groups were embedded in paraffin. The paraffin sections were stained with both Hematoxylin Eosin (HE) and Masson Trichrome (MT) according to standard methods and systematically scanned with a light microscope. These histological lung sections were used to determine lung inflammatory injury and lung fibrosis. 

### 4.5. Hydroxyproline Measurement 

The total collagen content of the right lung was determined by hydroxyproline assay kit. The right lung was vacuum-dried for 24 h using a Savant SpeedVac Concentrator (Thermo Fisher Scientific, Yokohama, Japan). After that, acid hydrolysis of the right lung was performed with 12 N HCL at 120 °C for 3 h in a Reacti-Vial (GL Sciences, Tokyo, Japan). The hydroxyproline content was determined using a Hydroxyproline Assay Kit (BioVision Research Products, Mountain View, CA, USA) according to the manufacturer’s instructions. 

### 4.6. BAL and Cell Counts in BALF

BAL was performed as described previously [19]. Briefly, the total number of cells in the BALF was counted with a hemocytometer. To obtain the BALF differential cell counts, Cytospin (Thermo Fisher Scientific, Yokohama, Japan) smear slides were prepared. The cell counts were obtained using standard light microscopy and staining with May-Gimza (Diff-Quik, Sysmex, Kobe, Japan). Differential cell counts were performed on 200 cells per smear. 

### 4.7. Measurement of the BALF Cytokine Concentration 

The concentration of MIP-2 (CXCL2), TNF-α, and TGF-β1 in the BALF supernatant was determined using an enzyme-linked immunosorbent assay (ELISA) kit according to the manufacturer’s instructions (R&D Systems, Minneapolis, MN, USA).

### 4.8. Quantitative Real-Time Reverse Transcription-Polymerase Chain Reaction

Total RNA was extracted from each lung tissue specimen using ISOGEN (Nippon Gene, Tokyo, Japan) in accordance with the manufacturer’s instructions. Complementary DNA (cDNA) was synthesized using a kit (High-Capacity cDNA Reverse Transcription Kit with RNase inhibitor; Applied Biosystems, Foster City, CA, USA) and quantified with a sequence detector (7500/7500 Fast Real-Time PCR System; Applied Biosystems) using TaqMan Universal PCR Master Mix (Applied Biosystems) and the relevant primers (Applied Biosystems), including a β-actin control. The mRNA expression levels of all samples were normalized to the level of the housekeeping gene β-actin. The names of the target genes and their assay IDs were as follows: β-actin: Mm00607939_s1; Heme oxygenase (HO)-1: Mm00516005_m1; NAD(P)H quinone dehydrogenase (NQO)1: Mm01253561_m1.

### 4.9. Statistical Analysis

Data are expressed as mean ± SD values. The Student’s *t*-test was used to determine the significance of differences between the groups, and *p*-values of <0.05 were considered to be significant.

## 5. Conclusions

DE is an important risk factor, and Nrf2 regulates the risk of a DE inhalation-induced immune response during BLM lung injury and fibrosis in mice.

## Figures and Tables

**Table 1:** The concentration of gases and particles in each chamber.

Chamber	CO (ppm)	SO_2_ (ppb)	NO (ppm)	NO_2_ (ppm)	NO_x_ (ppm)	DEP (mg/m^3^)	DEP# /cc
Clean	0.44 ± 0.17	0.64 ± 0.50	0.00 ± 0.01	0.02 ± 0.01	0.02 ± 0.01	0.01 ± 0.01	3 ± 1
DE	10.26 ± 2.72	21.03 ± 5.50	3.65 ± 0.84	1.91 ± 0.45	5.55 ± 1.26	1.02 ± 0.29	343,700 ± 2900

The values are mean ± SD of data in each group (*n* = 40 days). DEP #: DEP number.

## Supplementary Materials

Supplementary materials can be found at www.mdpi.com/1422-0067/18/3/649/s1.
