# Supplementary material for: Nrf2 Regulates the Risk of a Diesel Exhaust Inhalation-Induced Immune Response during Bleomycin Lung Injury and Fibrosis in Mice
Source: Int J Mol Sci. 2017 Mar 17;18(3):649. doi: 10.3390/ijms18030649 (PMC5372661; doi:10.3390/ijms18030649)
Supplement: Supplementary file 1 [file ijms-18-00649-s001.pdf]

Supplementary Materials: Nrf2 Regulates the Risk of a Diesel Exhaust Inhalation-Induced Immune Response During Bleomycin Lung Injury and Fibrosis in Mice

Ying-Ji Li, Takako Shimizu, Yusuke Shinkai, Yukiyo Hirata, Hirofumi Inagaki, Ken Takeda, Arata Azuma, Masayuki Yamamoto and Tomoyuki Kawada

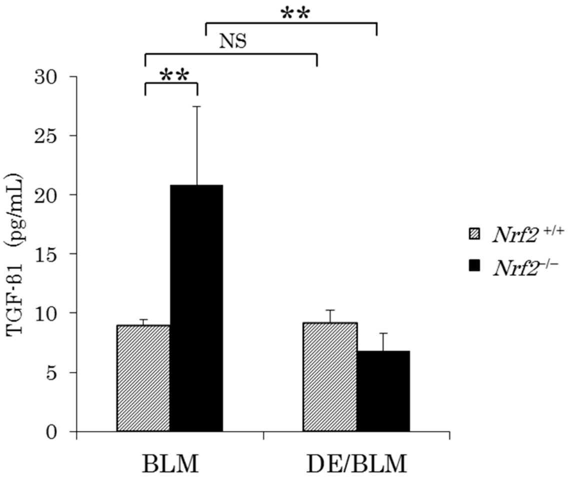

Figure S1. Comparison of transforming growth factor (TGF)-β1 content of lung tissues on day 10 after bleomycin (BLM) injection and after exposure to diesel exhaust (DE) for 38 days in both *Nrf2*$^{+/+}$ and *Nrf2*$^{-/-}$ mice. Data are shown as mean ± standard deviation (SD) values in each group. The significance of the interaction among the different groups was assessed using the analysis of variance (ANOVA), ** *p*-values < 0.001. NS: no significant difference.

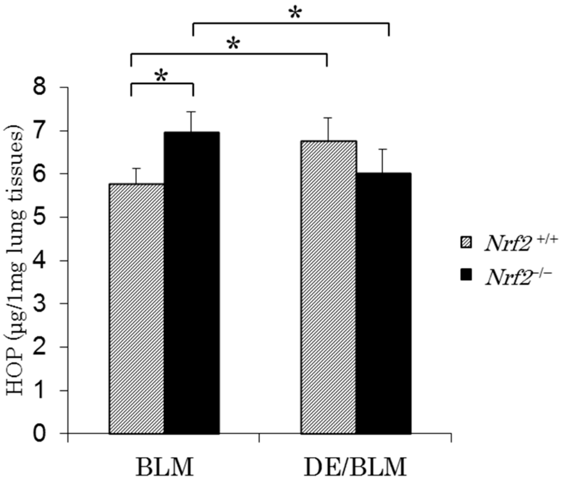

Figure S2. Hydroxyproline (HOP) content of lung tissues on day 28 after BLM injection and after exposure to DE for a maximum of 56 days in both *Nrf2*$^{+/+}$ and *Nrf2*$^{-/-}$ mice. Data are shown as mean ± SD values in each group. The significance of the interaction among the different groups was assessed using the analysis of variance (ANOVA), * *p*-values < 0.05.

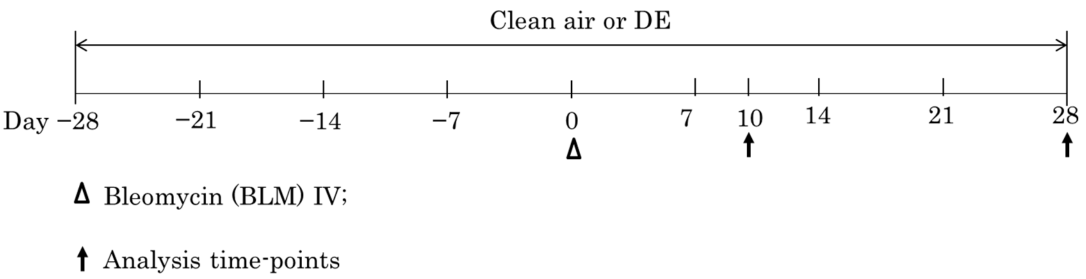

Figure S3. The experimental design is shown. Mice were exposed to DE or clean air for a maximum of 56 days. BLM was administered intravenously (IV) to mice on day 0. The analyses were performed 10 days after BLM injection for the BALF and for induction of mRNA of target genes in lung tissues. The analyses were performed 28 days after BLM injection for histopathological features and hydroxyproline measurement of the lung tissues.
